# Thrombocytosis portends adverse prognostic significance in patients with stage II colorectal carcinoma

**DOI:** 10.12688/f1000research.4856.2

**Published:** 2014-10-20

**Authors:** Tianhua Guo, Marcin Krzystanek, Zoltan Szallasi, Arpad Szallasi

**Affiliations:** 1 Department of Pathology, Monmouth Medical Center, Long Branch, NJ, NJ 07740, USA; 2 Department of Systems Biology, Technical University of Denmark, Lyngby, 2800, Denmark; 3 Children’s Hospital Informatics Program at the Harvard–Massachusetts Institute of Technology Division of Health Sciences and Technology, Harvard Medical School, Boston, MA, MA 02115, USA

## Abstract

Thrombocytosis portends adverse prognostic significance in many types of cancers including ovarian and lung carcinoma. In this study, we determined the prevalence and prognostic significance of thrombocytosis (defined as platelet count in excess of 400 × 10 ^3^/μl) in patients with colorectal cancer. We performed a retrospective analysis of 310 consecutive patients diagnosed at our Institution between 2004 and 2013. The patients (48.7% male and 51.3% female) had a mean age of 69.9 years (+/- 12.7 years) at diagnosis. Thrombocytosis was found in a total of 25 patients, with a higher incidence in those with stage III and IV disease (14.4% of patients). Although the mean platelet count increased with the depth of tumor invasion (pT), its values remained within normal limits in the whole patient cohort. No patient with stage I cancer (n=57) had elevated platelet count at diagnosis. By contrast, five of the 78 patients (6.4%) with stage II cancer showed thrombocytosis, and four of these patients showed early recurrence and/or metastatic disease, resulting in shortened survival (they died within one year after surgery). The incidence of thrombocytosis increased to 12.2% and 20.6%, respectively, in patients with stage III and IV disease. The overall survival rate of patients with thrombocytosis was lower than those without thrombocytosis in the stage II and III disease groups, but this difference disappeared in patients with stage IV cancer who did poorly regardless of their platelet count. We concluded that thrombocytosis at diagnosis indicates adverse clinical outcome in colorectal cancer patients with stage II or III disease. This observation is especially intriguing in stage II patients because the clinical management of these patients is controversial. If our data are confirmed in larger studies, stage II colon cancer patients with thrombocytosis may be considered for adjuvant chemotherapy.

## Introduction

Platelets play important roles in hemostasis, immunity and inflammation ^1^. Reactive thrombocytosis is an elevated platelet count (> 400 × 10 ^3^/μl) that develops secondary to another disorder. Some causes of reactive thrombocytosis include iron deficiency, acute infection, and chronic inflammatory disorders ( http://www.patient.co.uk/doctor/thrombocytosis). Cancer is often associated with paraneoplastic thrombocytosis ^2^. Thrombocytosis was reported as a poor prognostic indicator in many types of cancers including lung cancer ^3^, renal cell carcinoma ^4^ and gynecological cancers ^5^. A positive correlation between the depth of tumor invasion and platelet counts was demonstrated in a gastric cancer study, and thrombocytosis served as an adverse prognostic factor in clinical outcome in gastric cancer patients ^6^. Recent studies have shown that thrombocytosis in cancer may be correlated with serum cytokine levels that stimulate thrombopoiesis. For example, elevated plasma levels of IL-6 and thrombopoietin were reported in ovarian cancer patients ^5^.

Colon cancer is a leading cause of cancer-related death in developed countries ( http://seer.cancer.gov/statfacts/html/colorect.html). A significant portion of patients receiving potentially curative resection dies within five years of diagnosis ^7^. Since both chemo- and radiation therapies cause very significant side-effects, it is critical to define reliable prognostic factors to identify patients who might benefit from more aggressive adjuvant treatment options. The prognostic role of thrombocytosis in colorectal cancer patients has not been fully investigated, although there are some reports supporting the negative impact of thrombocytosis on the survival of patients with colorectal cancer ^8, 9^.

In this study, the aim was to analyze the association between platelet count at diagnosis or pre-surgery and cancer stage, and to determine the prognostic significance of thrombocytosis in patients with colorectal cancer.

## Patients and methods

After approval by the Monmouth Medical Center Institutional Research Review Board (IRB Study # 213-041), the medical records of 310 consecutive colorectal cancer patients who underwent biopsy (47 patients), surgical resection and/or neoadjuvant treatment (263 patients) at our Institution between 2004 and 2013 were retrospectively reviewed. The patient cohort included 62 patients with *in-situ* or stage I disease, 78 patients at stage II, 98 patients at stage III, 34 patients at stage IV, and 38 patients with biopsy diagnosis only (stage could not be determined). The mean age of the patients was 69.9 ± 12.7 years (range = 32 to 98 years). The data gathered included platelet counts, tumor location, histological type and differentiation, lymph node metastasis, depth of tumor invasion (T), and presence or absence of distant metastasis. The database did not include ethnicity, smoking habits, and comorbidities.

Pre-operative platelet counts were collected from our Laboratory Information System (LIS), and thrombocytosis was defined as platelet count ≥ 400 × 10 ^3^/μl (normal range = 150 to 400 × 10 ^3^/μl). After resection of the tumor, all specimens were histologically examined by a pathologist and the pathological TNM stage was determined according to the American Joint Committee on Cancer, 7 ^th^ edition. Stage I and II cancers are lymph node negative (N0) whereas stage III is defined by the presence of lymph node metastasis. Patients with distant metastatic disease are classified as stage IV.

Survival data for 253 patients were provided by the Cancer Registry at the Leon Hess Cancer Center, Monmouth Medical Center (57 patients were lost to follow-up). All calculations were made using R version 2.15.0 and packages “beeswarm”, “survplot”, “survival” and “stats”. Survival curves were generated using the Kaplan-Meier method. Hazard ratios with 95% confidence intervals were obtained using Cox proportional hazards regression. Long-rank test was used for the analysis of significance.

## Results

The characteristics (including age, gender, pT stage, tumor differentiation and platelet count) of the patients in our study cohort are shown in Table 1. Of the 310 patients with colorectal cancer, 25 (8.1%) had thrombocytosis at diagnosis or pre-surgery with the highest incidence detected in stage IV patients (20.6%) [ Table 2]. Importantly, none of the 57 patients with stage I carcinoma had elevated platelet count. The mean platelet counts (× 10 ^3^/µL) in patients with or without thrombocytosis were 494.7 ± 83.0 and 246.2 ± 72.3, respectively.

**Figure 1.**
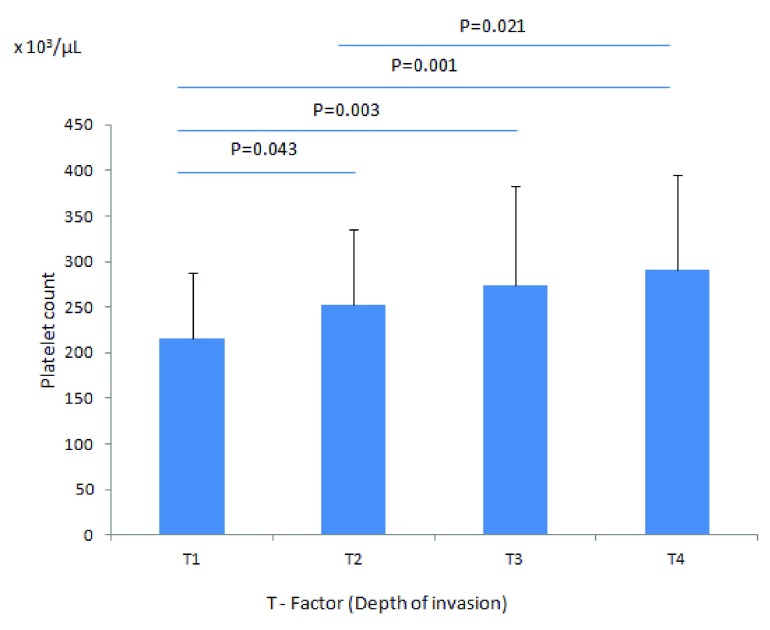
Mean platelet count according to the depth of tumor invasion. The mean platelet count of patients with pT1, (216 ± 71) × 10 ^3^/µL is significantly lower than patients with pT4 (291 ± 104) × 10 ^3^/µL, P = 0.001.

Although the mean platelet count increased with the depth of invasion (pT), it remained within the normal limits in all patients groups (pT1 to pT4) [ Figure 1]. Mean platelet counts (× 10 ^3^/µL) were 216 ± 71 (pT1), 252 ± 83 (pT2), 274 ± 109 (pT3), and 291 ± 104 (pT4); this increase was significant at P = 0.001. By contrast, in multivariate analysis, there were no significant differences in thrombocytosis with regard to gender, age, location, or tumor differentiation (data not shown). For example, our database included 193 patients with right-sided (from cecum to splenic flexure) and 102 patients with left-sided (from splenic flexure to rectosigmoid colon) cancer. Furthermore, we had one patient with rectal adenocarcinoma and 14 patients with metastatic (stage IV) colorectal carcinoma of unknown (at least to us) primary location. Fourteen of the 193 patients with right-sided (~7%) and 8 of the 102 patients with left-sided (~7%) carcinoma showed thrombocytosis at diagnosis, indicating that thrombocytosis is not predictive of the location of the tumor (right- *versus* left-sided).

**Table 1.** Patient characteristics.

	Number (%)
Total	310
Gender	
Male	151 (48.7%)
Female	159 (51.3%)
Age	69.9 ± 12.7 years ^a^
Pathologic category of primary tumor (pT) ^b^	
pTis	5 (1.6%)
pT1	17 (5.5%)
pT2	60 (19.4%)
pT3	137 (44.2%)
pT4	44 (14.2%)
pTx (unclassified)	47 (15.2%)
Grade ^c^	
well differentiated	42 (13.5%)
moderately differentiated	149 (48.1%)
poorly differentiated	67 (21.6%)
unclassified	52 (16.8%)
Platelet count (× 10 ^3^/µL)	
≥ 400	25 (8.1%)
< 400	285 (91.9%)

a: Data presented as mean ± SD; b: staging according to AJCC Cancer Staging Manual 7 ^th^ Edition); c: Grading according to WHO grading system

**Table 2.** Incidence of thrombocytosis in each stage of colorectal cancer.

	Number of cases with thrombocytosis	Number of cases without thrombocytosis	Total number of cases	% of thrombocytosis
Stage 0	0	5	5	0.0
Stage I	0	57	57	0.0
Stage II	5	73	78	6.4
Stage III	12	86	98	12.2
Stage IV	7	27	34	20.6
Stage X (unclassified)	1	37	38	2.6
Overall	25	285	310	8.1
*Stage IV and III combined	19	113	132	14.4

Note: There are 403 cases in our data pool. 310 cases were diagnosed as colon cancer among the 321 cases with available platelet counts (82 cases without platelet counts). 11 non-colorectal cancer cases including 5 cases labeled as “appendix” and 6 cases labeled as “not colon cancer” were taken out of the pool.

**Figure 2.**
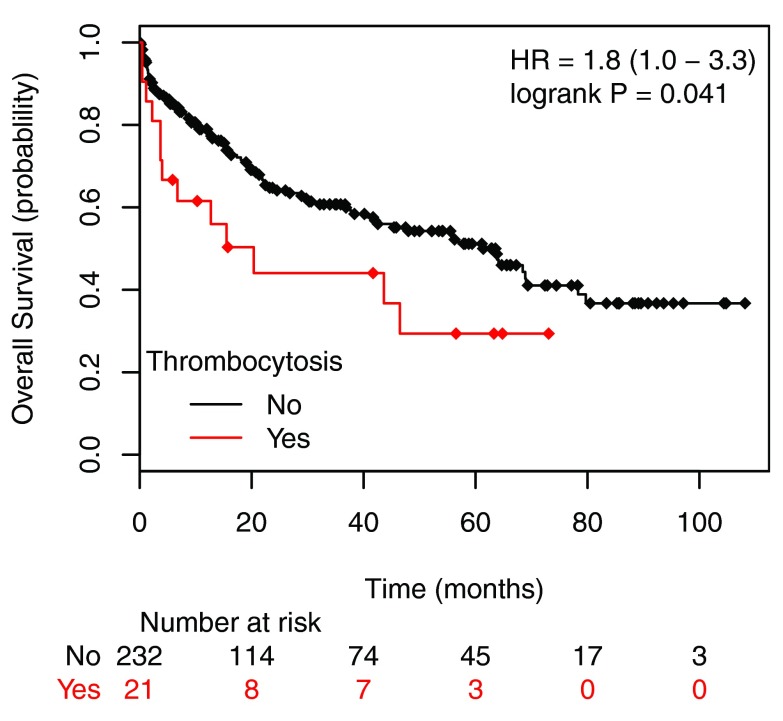
Overall survival plots of patients with stage I to stage III colorectal cancer according the preoperative platelet counts. Patients were allocated into two groups: with thrombocytosis, PLT ≥ 400 × 10 ^3^/µL (labeled as Exceeds) and without thrombocytosis) PLT < 400 × 10 ^3^/µL (labeled as Normal). The patients with thrombocytosis have a hazard ratio of 1.8 compare to the patients without thrombocytosis (P<0.041).

The combined incidence of thrombocytosis in stage III and IV disease was 14.4%. Stage II disease had the lowest incidence (6.4%) and stage IV cancer showed the highest incidence of elevated platelet count (20.6%) [ Table 2]. The overall survival of patients with stage I to stage III colorectal cancer with thrombocytosis was significantly lower than those without thrombocytosis [ Figure 2]. Although patients with Stage IV carcinoma had the highest prevalence of thrombocytosis, these patients did uniformly poorly and the difference in survival was no longer observed in patients with or without elevated platelet count (not shown). Patients with thrombocytosis (PLT ≥ 400 × 10 ^3^/μL) at stage I to stage III had a hazard ratio of 2.2 compared to the patients without thrombocytosis (PLT < 400 × 10 ^3^/μL) as shown in Figure 2.

Data of thrombocytosis in colon cancer patients This data set contains the medical records of patients diagnosed with colon cancer at the Monmouth Medical Center between 2004 and 2013.

## Discussion

It was noted more than 100 years ago that thrombocytosis is often seen in patients with malignant diseases. Indeed, thrombocytosis correlates with both worse disease free survival and shortened overall survival in patients with ovarian cancer ^5^, and worsens overall survival in patients with gastric cancer (reviewed in 10). The prognostic significance of thrombocytosis in colorectal cancer, however, remains controversial ^11^, although the majority of literature suggests a negative impact of thrombocytosis on the survival of patients with colorectal cancer ^8, 9^.

In the present study, we identified a mild, T stage-dependant increase in mean platelet counts in patients with colorectal carcinoma that reached statistical significance when comparing T1 to T4; however, the mean platelet count remained within the normal limits in the whole patient cohort. Importantly, thrombocytosis was more common among patients with advanced disease: its prevalence increased from 6.4% in stage II to 20.6% in stage IV cancer patients. Our data are comparable to those reported in the literature (12.1–13.9%; ^7, 8^).

In our study, thrombocytosis showed adverse prognostic significance in patients with stage I to stage III colorectal carcinoma; this was no longer apparent in patients with stage IV disease, presumably because these patients did poorly regardless of the platelet count.

Probably the most intriguing observation of our study is the fairly uniformly dismal clinical outcome of stage II patients with thrombocytosis (five out of 78 patients): four of these five patients did very poorly (they died within a year) and the fifth was lost to follow-up. At present, the National Comprehensive Cancer Network guidelines do not recommend routine administration of adjuvant chemotherapy to stage II colorectal cancer patients whose cancer was completely resected ^12^. Clearly, the small number of patients in this group precludes a definitive conclusion. However, if confirmed in future larger studies, our data indicate that stage II colorectal cancer patients with thrombocytosis may benefit from adjuvant chemotherapy.

The molecular mechanisms underlying thrombocytosis in cancer patients are incompletely understood. Recently, plasma levels of IL-6 and thrombopoietin were found to significantly correlate with platelet counts in ovarian cancer patients ^5^. Indeed, silencing of the IL-6 and thrombopoietin genes markedly abrogated thrombocytosis (and halted tumor progression) in a mouse model of epithelial ovarian cancer ^5^. These findings are promising because an anti-IL-6 agent (siltuximab) has already been approved by the United States Food and Drug Administration (FDA) to treat patients with Castleman’s disease ( http://www.cancer.org//cancer/new/fda-approves-sylvant-siltuximab-for-castleman-disease). Another series of study showed that immobilized platelets support human colon carcinoma cell tethering, rolling, and firm adhesion under dynamic flow conditions in colon cancer cell lines, suggesting a role of platelets in hematogenous dissemination of tumor cells in colorectal cancer ^13^. Taken together, these studies imply that inhibition of thrombocytosis may represent a potential novel target in cancer therapy.

In summary, thrombocytosis appears to herald adverse clinical outcome in patients with stage II and III colorectal carcinoma. We suggest that elevated platelet count may identify a subset of patients with stage II colon cancer who could benefit from close follow-up and adjuvant chemotherapy.

## Data availability

*F1000Research*: Dataset 1. Data of thrombocytosis in colon cancer patients, 10.5256/f1000research.4856.d33371 ^14^
