# Supplementary material for: Thrombocytosis portends adverse prognostic significance in patients with stage II colorectal carcinoma
Source: F1000Res. 2014 Oct 20;3:180. Originally published 2014 Jul 31. [Version 2] doi: 10.12688/f1000research.4856.2 (PMC4240242; doi:10.12688/f1000research.4856.2)
Supplement: Peer review history

# Peer review: Referee response for version 2

**DOI:** 10.5256/f1000research.5910.r6678

**Published:** 2014-11-10

**Authors:** Ioannis A. Voutsadakis

**Affiliations:** 1 Medical Oncology, Sault Area Hospital, Ontario, Canada

The report is now acceptable for indexing.

I have read this submission. I believe that I have an appropriate level of expertise to confirm that it is of an acceptable scientific standard.

# Peer review: Referee response for version 2

**DOI:** 10.5256/f1000research.5910.r6456

**Published:** 2014-10-27

**Authors:** Judit Moldvay

**Affiliations:** 1 Department of Pulmonology, Semmelweis University, Budapest, Hungary

The Authors have corrected the manuscript according to the referees’ comments and completions, therefore, this revised manuscript is acceptable for indexation.

I have read this submission. I believe that I have an appropriate level of expertise to confirm that it is of an acceptable scientific standard.

# Peer review: Referee response for version 1

**DOI:** 10.5256/f1000research.5184.r6229

**Published:** 2014-10-06

**Authors:** Judit Moldvay

**Affiliations:** 1 Department of Pulmonology, Semmelweis University, Budapest, Hungary

It is an interesting work providing new information on the issue of thrombocytosis in colon cancer patients. It contains relevant clinicopathologic parameters, however, there is no data on ethnicity, smoking habits, inflammatory processes, comorbidities, or medications of the studied patients, all which might have impact on platelet count (Msaouel *et al.*, 2014).

The main factors that can influence the platelet count should at least be discussed.

In Figure 1. the Authors discussed the mean platelet count according to the depth of tumor invasion. Although it might change statistically significantly, the mean platelet count remains much within the normal range, therefore, the conclusion that “… we identified a mild, T stage-dependant increase in mean platelet count …” is not well-founded. 

One of the most critical aspects of the investigation is the number of patients, especially in stage II, as disproportionately firm conclusions have been drawn regarding correlation between overall survival and thrombocytosis on the basis of only four patients with thrombocytosis in stage II.

Another weakness of the study is the lack of information on the precise localizations of the primary tumors, as this in itself may be of prognostic value, e.g. Majek *et al. *(2012) .

Although neoadjuvant treatment has been mentioned, it was not discussed in details, similarly, adjuvant treatments in advanced cases were not detailed either.These issues should be discussed in the "Discussion".

In Figure 2. the value of HR is not in accordance with the HR in the legend.

In the 4 ^th^ line of the Discussion there is a mistake with the Ref. 3., this – most probably – is the Ref. 5.

Similarly, in the 18 ^th^ line of the Discussion the Ref. 6. – most probably – is the Ref. 8.

In conclusion, this study yielded clinically very interesting results that might have therapeutic consequence, however, these results need to be confirmed in a much larger cohort of colorectal patients.

All in all, in my opinion this manuscript is acceptable for indexation, but only after corrections and completions based on the above mentioned comments.

I have read this submission. I believe that I have an appropriate level of expertise to confirm that it is of an acceptable scientific standard, however I have significant reservations, as outlined above.

# Response

**Published:** 2014-10-13

**Authors:** Arpad Szallasi

**Affiliations:** Department of Pathology, Monmouth Medical Center, USA

Whereas we share some of the reservations of the referee, we would like to point out that this is an Observational Study and not a full report. We believe that despite the limitations of this study, this is an interesting observation with potential implications for patient management. That said, we wholeheartedly agree with the referee that "these results need to be confirmed in a much larger cohort of colorectal patients." 

Changes done in response to points raised by the referee in the revised MS include:

1. Our study has a number of limitations such the lack of data on ethnicity, smoking habits, comorbidities, or medications: this is now clearly stated in Patients and Methods. We believe that such a detailed, multifactorial analysis would surpass the confines of a preliminary Observational Report. Of note, the patient database is available for review for the interested reader (please follow link under Reference 14 to "data source").
2. The most common causes of reactive thrombocytosis are now briefly listed in the Introduction.
3. The statement between platelet count and depth of invasion has been toned down - the reference to significance has been eliminated.
4. As emphasized in the MS, our conclusions are preliminary and are based on a limited number of patients. It is our hope that our findings will be confirmed (or refuted) based on a much larger patient cohort. 
5. The localization of the primary tumor (right-sided versus left-sided) seems to have no impact on the presence or absence of thrombocytosis: both groups had an incidence of ~7%. This is now clearly stated in Results. 
6. References have been corrected.

# Peer review: Referee response for version 1

**DOI:** 10.5256/f1000research.5184.r5655

**Published:** 2014-08-14

**Authors:** Ioannis A. Voutsadakis

**Affiliations:** 1 Medical Oncology, Sault Area Hospital, Ontario, Canada

This is an interesting retrospective study of an extensive series of colorectal cancer patients and authors are to be congratulated on undertaking this academic endeavour. Nevertheless there are several points that need to be addressed. A major concern is that all stages of the disease have been included. In general a comparison between stage I and IV, for example, has no meaning clinically and the only statement that one can make is that thrombocytosis is more common. Another major concern is that no multivariate analysis is done or presented.

Some other specific points:

- The title mentions specifically stage II disease (as the authors probably recognize the importance of prognostic factors in this sub-group for therapeutic decisions) but no statistical comparison is offered anywhere in the article. In any case the number of patients with thrombocytosis in this group (5) is prohibitive for definite conclusions.
- In the last line of the Abstract and in the Discussion it should be: “ *treated as stage III*” or even better “ *considered for adjuvant chemotherapy*” (stage IV patients are treated with palliative intent).
- Data on tumor grade and location have been collected (dataset) but not summarized or presented in the article. It would be of particular interest to present and include in an eventual multivariate analysis tumor location (right versus left from the splenic flexure versus rectal).
- In Patients and methods the normal range of platelets in authors’ institution should be mentioned.
- In the Results it would be interesting to provide the mean and SD of the platelet number in the 2 groups (normal and thrombocytosis).
- In fig. 2 there is a discrepancy between HR and p values in the body and the legend. In addition the numbers at risk do not match. Is that a mistake in calculations or data missing? Authors should address this.
- In the 4 ^th^ line of the 1 ^st^ paragraph of the Discussion” the reference does not seem to be correct. Same with reference 6 in the 2 ^nd ^paragraph.
- In the discussion on metastatic patients - this is an over-simplification. If any useful comparison is to be made, other data have to be entered in the equation such as oligometastatic versus polymetastatic disease and treatments.
- Treatments should be mentioned. For example have all stage III patients received adjuvant chemotherapy? And have any of the stage II patients received any such treatment?
- In the 4 ^th^ paragraph of the Discussion the explanation of “ *false negative node examination*” is improbable as there is no reason to suspect that such false negative occurs more commonly in patients with thrombocytosis. Instead a difference in biology as briefly discussed in the next paragraph is more probable.

I have read this submission. I believe that I have an appropriate level of expertise to confirm that it is of an acceptable scientific standard, however I have significant reservations, as outlined above.

# Response

**Published:** 2014-10-13

**Authors:** Arpad Szallasi

**Affiliations:** Department of Pathology, Monmouth Medical Center, USA

We are grateful for the referee for his thoughts in making this a better MS. Whereas we agree with most of the reservations of the referee, we would like to point out that this is an Observation Study, and not a full report. We believe that despite the limitations of our study, our conclusions are sound and (if confirmed) may have important implications for patient management.

Major changes done in response to points raised by the referee include:

1. We agree with the referee that the small number of patients (5) in the stage II group precludes a definitive conclusion; this is now clearly stated in the MS. Again, this is an Observational Study within the confines of our hospital database on colorectal carcinoma patients.
2. Both the Abstract and the Discussion have been modified as suggested by the referee ("treated as stage III" has been changed to "may be considered for adjuvant chemotherapy").
3. The multivariate analysis with special regard to the location of the tumor (right-sided versus left-sided) in now detailed in the Results.
4. The normal range of platelets at our Institution (150 to 400 K/ul) is now provided in the MS.
5. The mean +/-SD of the platelet counts in the two patient groups (with and without thrombocytosis) is now provided in Results.
6. Indeed, there is a discrepancy between the body of the text and Figure 2 with regard to the patient numbers; this should have been addressed before to avoid any confusion. As clearly stated now in Patients and Methods, our database included 310 patients. Of these patients, our Cancer Registry had long-term survival data on 253 patients (hence the discrepancy): the remaining 57 patients most likely decided to seek treatment at a different facility.
7. References have been corrected.
8. Discussion on metastatic patients has been modified.
9. Unfortunately, our Cancer Registry has no full details on chemotherapy regimens (some of our patients receive chemotherapy in a private practice setting).
10. The reference to "false negative node examination" has been eliminated.
